# The effects of transcranial direct current stimulation on global cognitive function, visuospatial function, and executive function in patients with mild cognitive impairment and Alzheimer’s disease: a meta-analysis

**DOI:** 10.3389/fneur.2026.1782579

**Published:** 2026-05-22

**Authors:** Fangzhou Yu, Mingchen Wang

**Affiliations:** 1 Cangzhou Hospital of Integrated Traditional Chinese and Western of Hebei Province, Cangzhou, Hebei, China; 2 Tianjin University of Traditional Chinese Medicine, Tianjin, China

**Keywords:** Alzheimer’s disease, global cognition, mild cognitive impairment, transcranial direct current stimulation, visuospatial function

## Abstract

**Background:**

Transcranial direct current stimulation (tDCS) has shown promise in improving the cognitive function of patients with Alzheimer’s disease (AD) and mild cognitive impairment (MCI). However, data from clinical trials or meta-analyses involving tDCS have produced conflicting results.

**Objective:**

This systematic review and meta-analysis aimed to evaluate the efficacy of tDCS in improving global cognition and specific cognitive domains in patients with AD and MCI.

**Methods:**

The review and analysis were conducted in accordance with the Preferred Reporting Items for Systematic Reviews and Meta-Analyses (PRISMA) guidelines. Four electronic databases—PubMed, Web of Science, the Cochrane Library and Embase—were searched up to 20 August 2025. Cochrane’s risk of bias assessment tools were used to evaluate the risk of bias and the methodological quality of the included studies. Statistical analyses were performed using Review Manager 5.3.

**Results:**

This meta-analysis of 13 studies (*n* = 616) showed that tDCS significantly improved global cognition as measured by MMSE and MoCA (SMD = 0.49). For other cognitive domains, evidence was limited: visuospatial ability (4 studies, Clock Drawing Test) showed a significant but preliminary effect (SMD = −0.75); attention (2 studies, Digit Span Test) showed a small effect (SMD = −0.33); executive function (4 studies, Trail Making Test) showed no significant effect (SMD = 0.09).

**Conclusion:**

tDCS significantly improves global cognition in patients with AD and MCI. Preliminary evidence suggests possible benefits for visuospatial ability, but findings for attention and executive function remain inconclusive. Future studies should employ multi-test, domain-specific neuropsychological batteries in adequately powered trials.

## Introduction

The most common forms of dementia in adults are progressive neurodegenerative disorders, such as Alzheimer’s disease (AD) and mild cognitive impairment (MCI). In 2010, it was estimated that 35.6 million people worldwide were living with AD or related dementias. With around 4.6 million new cases reported each year, this figure is expected to increase to 81.1 million by 2040 (1, 2). Current clinical guidelines recommend memantine, cholinesterase inhibitors and partial N-methyl-D-aspartate receptor antagonists as the primary treatment for patients at different stages of MCI and AD. However, the efficacy of these medications is limited and they are often associated with adverse side effects (3). There is an urgent need for alternative therapies, particularly since most forms of dementia, especially AD and MCI, still lack clearly defined and effective treatment strategies.

Transcranial direct current stimulation (tDCS) is a non-invasive neuromodulation technique that is renowned for being safe, well-tolerated, painless, affordable and easy to use. It also has the advantage of being able to be used at home, thereby improving accessibility for a broader patient population (4). These benefits have attracted growing clinical and scholarly interest in tDCS in recent years. The procedure involves applying two or more electrodes to the scalp to deliver a low-intensity current, typically ranging from 1 to 2 mA. As the current passes through the scalp, skull, and cerebrospinal fluid, it induces a weak electric field within the brain (5). tDCS was originally thought to act primarily through this electric field. Anodal stimulation depolarises and cathodal stimulation hyperpolarises neuronal membranes. However, emerging evidence suggests that tDCS may also modulate memory. It does this by stimulating cranial and cervical nerves in the scalp. These nerves relay signals to the brain. This promotes neuromodulation (6, 7).

Recent clinical trials have shown that tDCS may improve specific cognitive abilities in individuals with AD or MCI (8, 9). For example, single or repeated sessions of tDCS targeting the left dorsolateral prefrontal cortex (DLPFC) or the bilateral temporoparietal regions have been found to enhance cognitive performance in these patients (10, 11). Consistent with these findings, two meta-analyses have reported that anodal tDCS over the DLPFC, inferior frontal cortex or inferior frontal gyrus can improve cognitive function in healthy individuals, particularly when applied at a high current intensity and density (12, 13). Furthermore, studies suggest that tDCS may produce greater cognitive benefits in adults than in younger individuals (14)—an important consideration given that sporadic AD predominantly affects older populations. This age-related advantage may be due to lower baseline cognitive performance and greater potential for improvement in adults (15).

However, not all studies have found positive outcomes. Some studies have found that repeated tDCS applied to the left temporal lobe does not lead to significant cognitive improvement in patients with AD or MCI, suggesting that the results may not be applicable to everyone (16). A recent meta-analysis also concluded that tDCS has only minimal cognitive effects in individuals with MCI or AD (17). Systematic reviews and meta-analyses, as objective syntheses of existing evidence, help clarify discrepancies between preclinical and clinical findings. Although two earlier meta-analyses evaluated the effects of tDCS in patients with MCI, neither focused specifically on patients with AD nor were they based on data from patients with AD (18). To address this gap, the present study systematically reviews and meta-analyses the effects of tDCS on cognitive function in adults diagnosed with both AD and MCI.

## Methods

The review was published in compliance with the Preferred Reporting Items for Systematic Reviews and Meta-Analysis (PRISMA) criteria and was listed in the International Prospective Register of Systematic Reviews (PROSPERO: CRD42025633935).

### Search strategy

Two reviewers (MCW and FZY) independently searched the PubMed, Embase, Cochrane Library and Web of Science databases for studies containing the following keywords in their titles and abstracts: (a) transcranial direct current stimulation (tDCS) and transcranial electrical stimulation; (b) cognitive function; (c) Alzheimer’s disease (AD) and mild cognitive impairment (MCI). A senior researcher (GW) assessed the literature against the inclusion and exclusion criteria and made the final decision on the list of selected articles in cases of disagreement between the two reviewers. The search was conducted on 20 August 2025. No publication year filter was applied. The observed publication year range of the included studies (2014–2024) reflects the distribution of studies that met our eligibility criteria after screening, not an *a priori* restriction. No studies published in 2025 met the inclusion criteria at the time of the search.

### Eligibility criteria

The inclusion criteria were determined according to the PICOS methodology: (1) Patients diagnosed with MCI or AD according to Peterson’s MCI criteria (19), the Diagnostic and Statistical Manual of Mental Disorders, Fifth Edition (DSM-5) (20), or the NIA-AA criteria for AD (21); (2) Intervention comprising single or multiple sessions of tDCS applied to specific cortical regions; (3) Control group may receive Sham tDCS or a combination of Sham tDCS and physical training; (4) Study design was a Randomised Controlled Trial (RCT); (5) Articles were published in English. The exclusion criteria were as follows: (1) interventions other than tDCS; (2) studies published as conference proceedings; and (3) *in vitro* experimental studies.

### Data extraction

The data extracted from the final included studies comprised the following: the first author; the publication year; the country; the number of subjects; the disease type; the age; the educational attainment; the stimulus type and characteristics (including the current intensity, the single-stimulus duration, the stimulation site, the stimulation frequency and the duration); and the neurobehavioural outcomes (the mean results and the standard deviation (SD)). The primary outcome measures comprised global cognitive function, as assessed by the Mini-Mental State Examination (MMSE) or the Montreal Cognitive Assessment (MoCA). Secondary outcome measures included measures of visuospatial function (e.g., the clock-drawing test), executive function (e.g., the Trail Making Test Parts A and B), and attention (e.g., the digit span test, both forward and backward). Where a single study comprised multiple trials, the data were assessed as independent trials. Where multiple neurobehavioural outcomes were reported for the same subject, only the final assessment data were included. Within the same study, the anodal and cathodal phases of tDCS were considered separate trials.

### Quality assessment

The risk of bias was assessed using Cochrane guidelines and techniques (22). Two independent authors (MCW and FZY) used these techniques to evaluate various factors, such as random sequence generation, allocation concealment, blinding of participants and researchers, blinding of outcome assessment, incomplete or selective reporting, and other potential biases. Any disagreements between the two assessors were resolved by the senior author (GW). The risk of bias for each element was rated as low, unclear or high.

### Statistical analyses

All data from the RCTs and crossover studies included in the review were analysed using Review Manager (RevMan) software (version 5.4) for meta-analysis. The changes in outcome measures between the intervention and control groups after the intervention were expressed as mean differences with SD. Continuous variables were assessed using standardised mean difference (SMD) with 95% confidence intervals (CI). Heterogeneity across studies was assessed using the I^2^ statistic, with values of 25, 50 and 75% representing low, moderate and high heterogeneity, respectively. All meta-analyses employed a fixed-effects model, with *p* < 0.05 indicating statistically significant differences.

## Results

**Figure 1:**
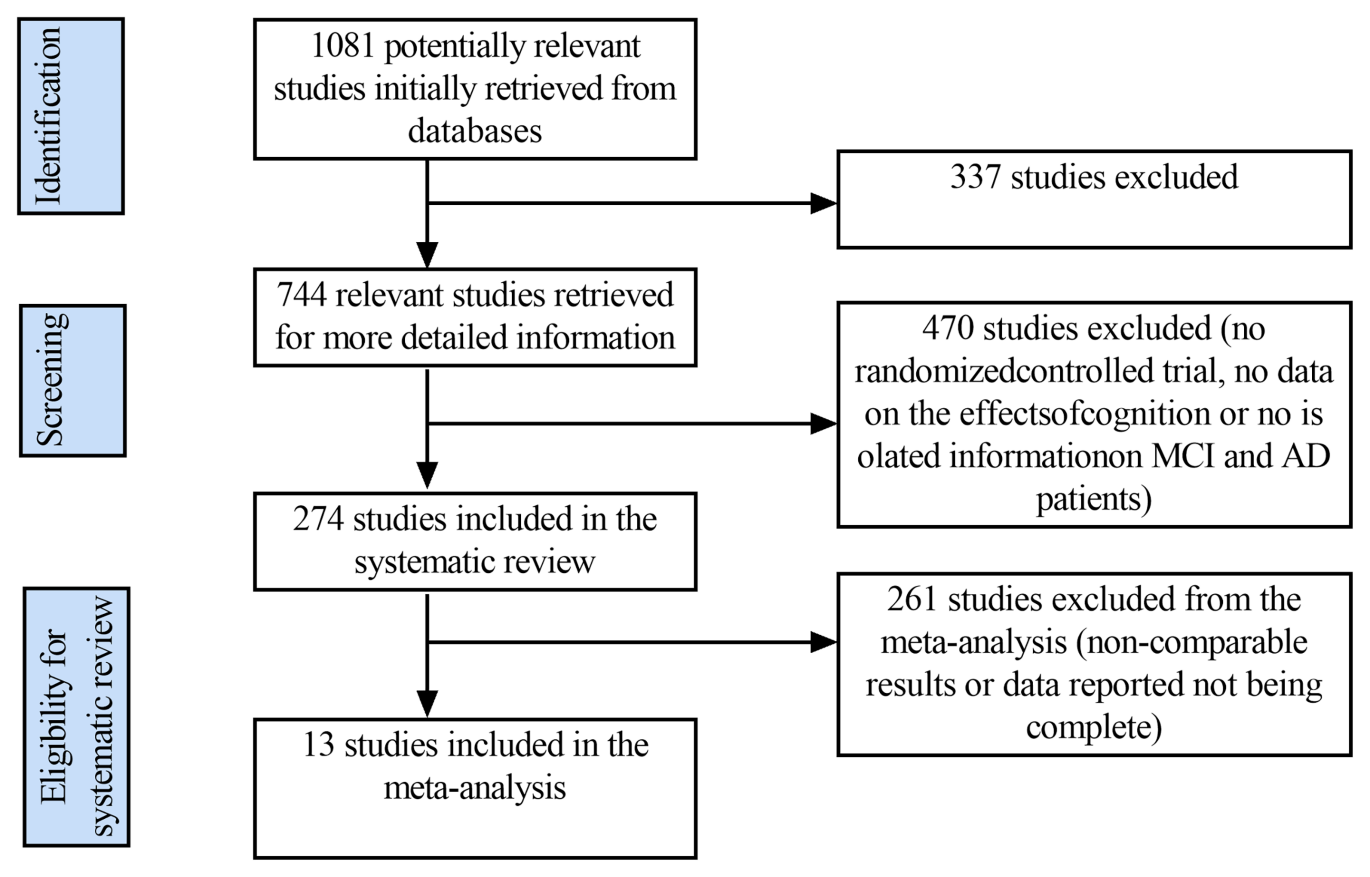
Literature screening flowchart.

The initial search yielded 1,081 results from the PubMed, Web of Science, Embase and Cochrane Library databases. After removing 337 duplicate publications, 744 papers remained. A further 470 studies were excluded (as they were non-RCTs or lacked cognitive function impact data or independent information on patients with MCIor AD), leaving 274 eligible papers. Subsequently, a further 261 studies were excluded from the meta-analysis due to a lack of comparability or incomplete data reporting. Ultimately, thirteen studies were included in the systematic review (Figure 1).

### General study characteristics

This review included 13 studies involving 616 participants. Six of these studies examined AD (16, 23–27), one study included both AD and MCI (28), and the remaining six studies focused on MCI (29–34). These studies investigated the effects of tDCS on cognitive function in individuals with AD or MCI. The studies were conducted between 2014 and 2024 in multiple countries, including Spain, China, Egypt, South Korea, Brazil, Norway, Iran, Japan, Italy and Hong Kong. The research design typically compared sham tDCS (the control group) with active tDCS, which was often combined with additional interventions such as cognitive training, Tai Chi, or working memory training. The stimulation parameters were largely consistent across the studies, with current intensities ranging from 1.5 to 2 mA and session durations ranging from 20 to 30 min. The DLPFC was the most frequently targeted site, though other brain regions such as the temporal lobe or bilateral DLPFC were also studied (Table 1).

**Table 1:** Characteristics of the studies included in the systematic review.

Author	Year	Country	C/T	Disease	Age C/T	Education years, C/T	Intervention, C/T	Intensity of Stimulation	Stimulation site	Frequency and duration	Global cognition	Visuospatial	Execution	Attention
Melendez et al. (23)	2023	Spain	9/9	AD	75.4 ± 4.4/73.7 ± 3.8	8.88 ± 4.6/7.88 ± 3.9	①/②	2 mA, 20 min	Left DLPFC	1sessions/d, 5d	MMSE			
Khedr et al. (24)	2019	Egypt	21/23	AD	65.23 ± 4.52/64.22 ± 3.64	3.52 ± 1.96/4.04 ± 2.83	①/②	2 mA, 20 min	Left temporal lobe and right temporal lobe	5 sessions/w, 2w	MMSE	Clock drawing test		
Bystad et al. (16)	2016	Norway	13/12	AD	70.0 ± 8.0/75.0 ± 8.7	–	①/②	2 mA, 30 min	Left temporal lobe	6 sessions for 10 days	MMSE	Clock-drawing test	TMT-A, TMT-B	
Lu et al. (25)	2019	China Hong Kong	64/69	AD	74.2 ± 6.7/74.5 ± 6.6	7.3 ± 4.8/6.5 ± 4.3	①⑤/②⑤	2 mA, 20 min	Left lateral temporal cortex	3 sessions per week for 4 weeks	MMSE		TMT-A, TMT-B	
Gangemi et al. (26)	2021	Italy	13/13	AD	69.01 ± 3.1/67. 5 ± 2.8	6.1 ± 2.1/6.5 ± 2.0	①/②	2 mA, 20 min	Left fronto temporal cortex	10 sessions each month for 8 months	MMSE			
Cotelli et al. (27)	2014	Italy	12/12	AD	74.7 ± 6.1/76.6 ± 4.6	8.9 ± 5.1/5.5 ± 2.4	①/②④	2 mA, 25 min	Left DLPFC	10 sessions over 2 weeks for 3 months	MMSE		TMT-A, TMT-B	
Rodella et al. (28)	2021	Italy	15/13	AD, MCI	75.13 ± 4.76/71.62 ± 5.65	9.67 ± 4.98/11.08 ± 4.99	①/②④	2 mA, 30 min	Left DLPFC	12 sessions over 3 weeks for 6 months	MMSE			
Xu et al. (29)	2023	China	49/44	MCI	61 ± 8.5/59 ± 8.75	9 ± 5/9.5 ± 3.75	①③/②③	2 mA, 20 min	Right DLPFC	5 sessions/w, 12w	MoCA			
Im et al. (30)	2019	South Korea	7/11	MCI	71.9 ± 9.2/74.9 ± 5.0	6.3 ± 3.8/5.4 ± 5.9	①/②	2 mA, 20 min	Left DLPFC	Daily, for 6 months	MMSE	Clock Drawing Test		
Gomes et al. (31)	2019	Brazil	29/29	MCI	71.6 ± 7.9/73.0 ± 9.2	–	①/②	2.0 mA, 30 min	Left DLPFC	Twice per week for 5 weeks	MMSE	Clock Drawing Test		
Gonzalez et al. (32)	2020	China Hong Kong	24/24/21	MCI	71.0 ± 6.2 69.8 ± 5.3/70.6 ± 5.4	9.7 ± 3.6/9.7 ± 3.6/11.9 ± 4.9	①④/②④	1.5 mA, 30 min	Left DLPFC	3sessions/w,3w	MoCA		TMT-A, TMT-B	DST backward, DST forward
Rezakhani et al. (33)	2024	Iran	20/20/20	MCI	69.35 ± 9.94/68.25 ± 10.26/69.05 ± 9.91	–	①/②	2 mA, 20 min	Left DLPFC and DATL	Duration 2 weeks (5 consecutive days per week for a total of 10 days)	MOCA			
Inagawa et al. (34)	2019	Japan	7/13	MCI	76.2 ± 7.7/76.6 ± 5.7	–	①④/②④	2 mA, 20 min	Left DLPFC	2 sessions per day for 5 consecutive days	MMSE			DST backward, DST forward

① sham; ② tDCS; ③ Taichi; ④ Cognitive training; ⑤ working memory training; DLPFC, dorsolateral prefrontal cortex; C, control group; T, treatment group.

### Risk of bias assessment

**Figure 2:**
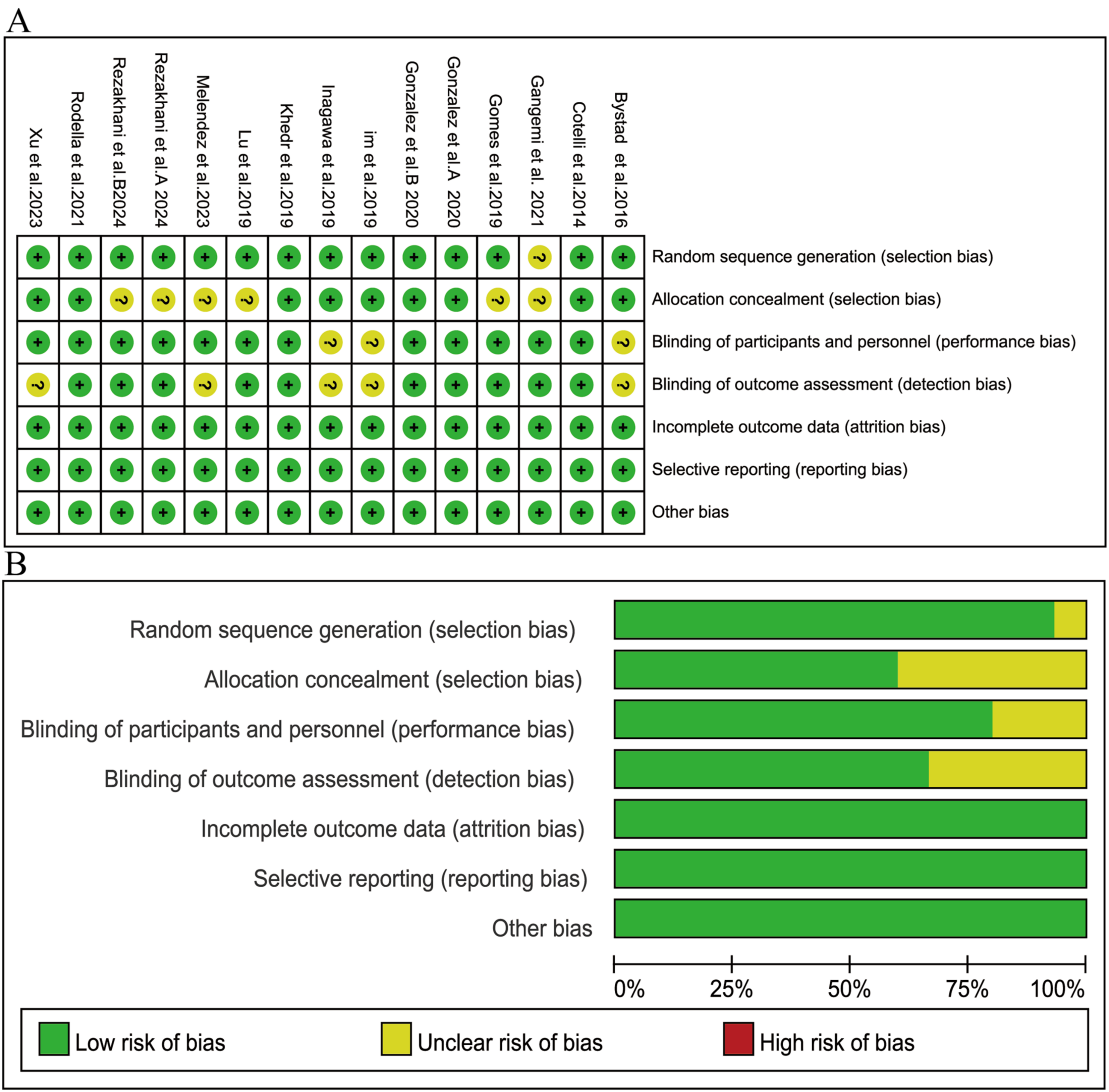
Article quality assessment. **(A)** Risk of bias summary; **(B)** Risk of bias graph.

Publication bias was identified using Cochrane’s risk-of-bias assessment techniques. All studies were published in peer-reviewed journals. The results indicated adequate reporting of outcome data, with no evidence of selection bias (reporting bias). However, random sequence generation was not observed in one study (26), blinding of participants and personnel was not observed in three studies (16, 30, 34), and allocation concealment was not observed in five studies (23, 25, 26, 31, 33). Overall, the quality of the included trials was good. The risk of bias assessment for the studies included in this review is presented in Figure 2.

### Synthesis

We conducted a meta-analysis across four distinct cognitive domains. 1. Global cognitive function (MMSE and MOCA), 2. Visuospatial function (Clock Drawing Test), 3. Executive function [Trail Making Test (TMT-A and TMT-B)], 4. Attention [Digit Sequence Test forward and backward (DST-forward and DST-backward)].

### Global cognitive function

**Figure 3:**
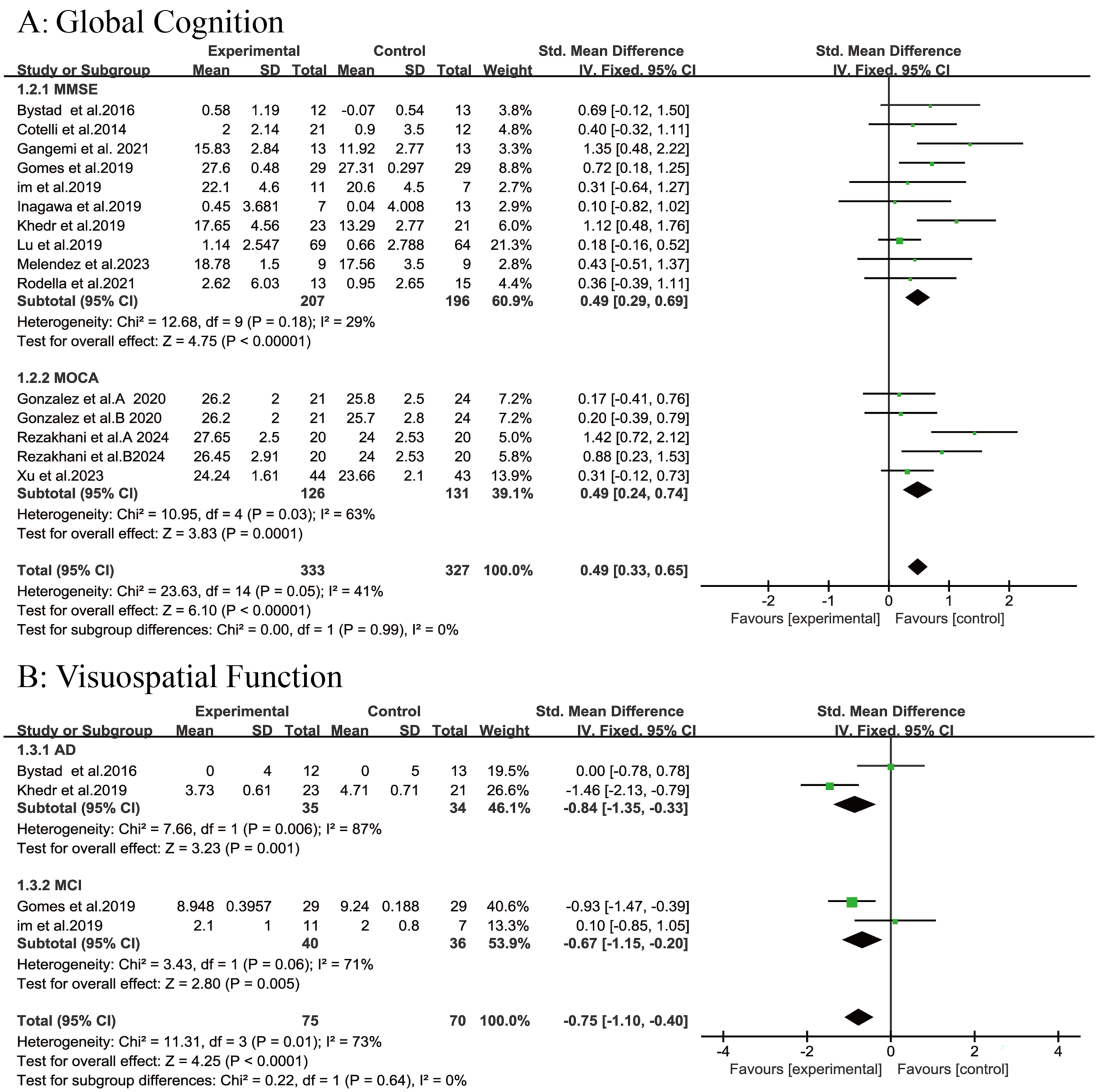
Forest plot. **(A)** Global cognition; **(B)** Visuospatial function.

Thirteen studies reported overall cognitive scores for a total of 616 patients, with ten of these studies employing the MMSE scale (*n* = 394) and three using the MOCA scale (*n* = 222). The results of the meta-analysis demonstrated that tDCS significantly enhanced overall cognitive scores in patients with AD and MCI compared with controls (SMD = 0.49, 95% CI (0.33, 0.65), *p* < 0.00001; Figure 3A). Subgroup analyses revealed that tDCS significantly improved MMSE cognitive scores in patients with AD and MCI (SMD = 0.49, 95% CI (0.29, 0.69), *p* < 0.00001; Figure 3), as well as MOCA cognitive scores (SMD = 0.49, 95% CI (0.24, 0.74), *p* = 0.0001; Figure 3A).

### Visuospatial function

A meta-analysis of visual–spatial function was conducted, including four studies involving a total of 145 patients (two studies focused on AD with 69 participants, and two studies focused on MCI with 76 participants). The results showed that tDCS was significantly more effective than the control treatment (SMD = −0.75, 95% CI [−1.10, −0.40], *p* < 0.0001; Figure 3B). Subgroup analyses confirmed significant improvements in both the AD group (SMD = −0.84, 95% CI [−1.35, −0.33], *p* = 0.001) and MCI group (SMD = −0.67, 95% CI [−1.15, −0.20], *p* = 0.005).

### Executive function

**Figure 4:**
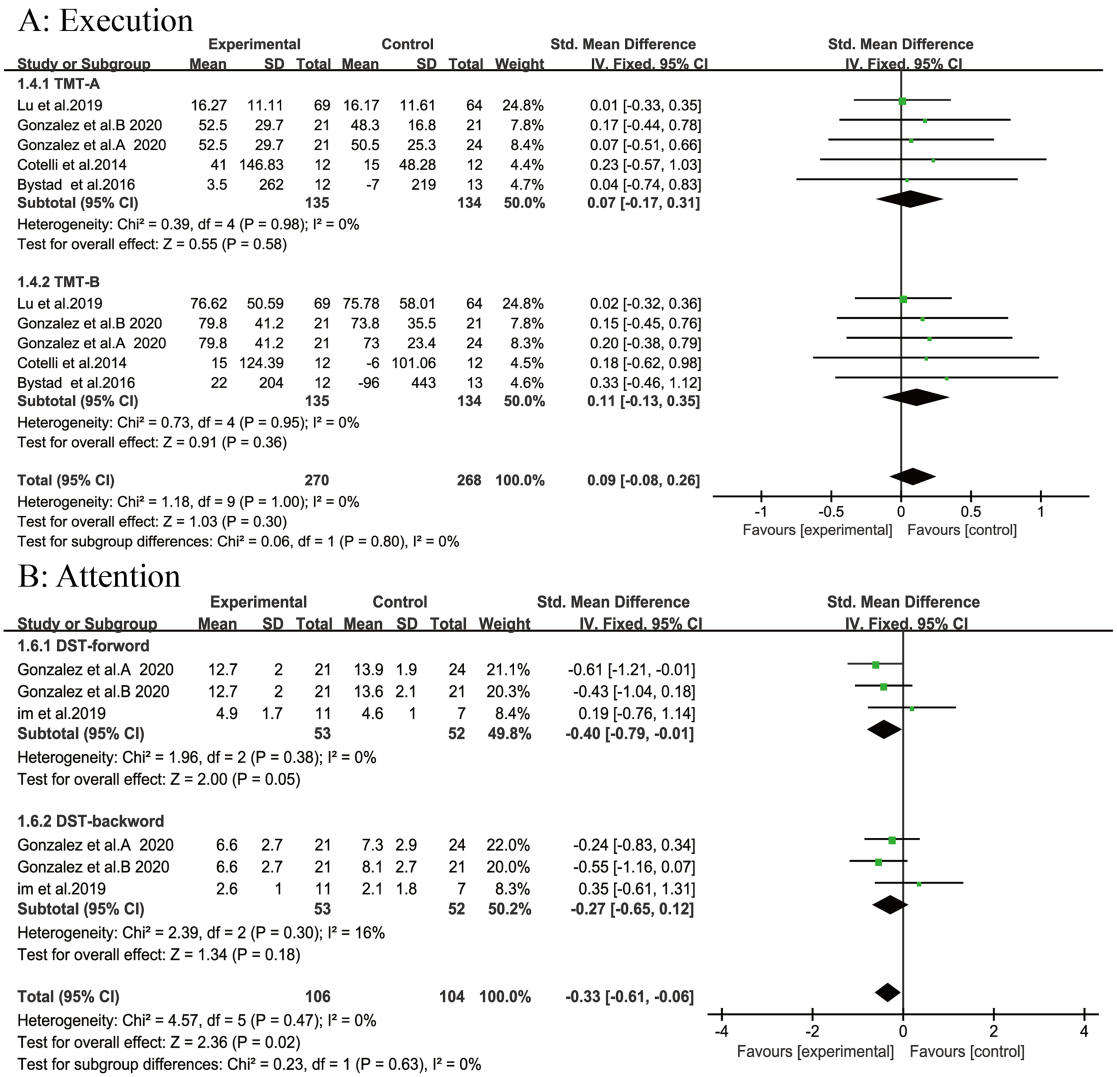
Forest plot. **(A)** Execution; **(B)** Attention.

A meta-analysis of four studies involving 251 patients demonstrated that tDCS had no significant effect on overall executive function in patients with AD or MCI (SMD = 0.09, 95% CI [−0.08, 0.26], *p* = 0.30; Figure 4A). Subgroup analyses revealed no significant effects on TMT-A (SMD = 0.07, *p* = 0.58) or TMT-B (SMD = 0.09, *p* = 0.36).

### Attention

Two studies reported attention scores for 87 patients. Our meta-analysis indicates that tDCS produces a statistically significant improvement in overall attention in patients with AD and MCI, as confirmed by pooled attention scores (SMD = −0.33, 95% CI (−0.61, −0.06), *p* < 0.02; Figure 4B). However, when we decomposed the attention construct into more specific subdomains via subgroup analysis, the results revealed more subtle differences. The effect on DST-forward scores approached, but did not quite reach, statistical significance (SMD = −0.40, *p* = 0.05). In contrast, the effect on DST-backward scores was not statistically significant (SMD = −0.27, *p* = 0.18; Figure 4B).

## Discussion

### Summary of the evidence

The meta-analysis of 13 relevant studies provides robust evidence that tDCS improves global cognition. Preliminary evidence suggests possible benefits for visuospatial ability, but findings for attention and executive function remain inconclusive.

### Compared with other studies

Current research findings regarding the effects of tDCS on cognitive deficits in AD or MCI are inconsistent. Two previous meta-analyses have examined the impact of tDCS on cognitive impairment in AD and MCI (35, 36). According to Saleh et al. (35) meta-analysis of 11 studies, tDCS did not improve overall functioning as measured by the MOCA. However, our study demonstrated that tDCS significantly enhances MOCA cognitive scores in patients with AD and MCI (SMD = 0.49, *p* = 0.0001), contradicting the former study’s findings. Furthermore, the limited number of studies and the use of multiple assessment measures in previous research make it difficult to draw definitive conclusions regarding the efficacy of multiple anodal tDCS sessions. A meta-analysis by Hou et al. (36), which examined the effects of tDCS on cognitive function in 36 studies, indicated that tDCS may improve overall cognitive function in patients with AD and MCI. However, the paper explicitly notes high heterogeneity in the pooled analyses of the MMSE and MoCA scores (I^2^ reaching 92 and 89%, respectively). Although the authors attempted to explore and reduce sources of heterogeneity through sensitivity and subgroup analyses (e.g., by intervention duration), the existence of such analyses indicates significant differences between the original studies. In contrast, our meta-analysis exhibits lower heterogeneity, suggesting good consistency amongst the results of the included studies. This enhances the reliability of our conclusions.

Importantly, of the six MCI studies included in our review, two (29, 33) were published after the search cut-off date of the most recent MCI-focused meta-analysis by Li et al. (18) search up to October 14, 2023, and thus represent new evidence not previously synthesised. Furthermore, unlike previous meta-analyses that focused solely on global cognition (MMSE/MoCA), the present study extracted and analysed domain-specific outcomes (e.g., visuospatial ability, attention, executive function) from the same studies, providing novel effect estimates that had not been reported previously.

Our research indicates that tDCS does not significantly improve executive function in patients with AD and MCI, particularly when the TMT-A and TMT-B serve as primary measurement indicators, consistent with the findings of Chen et al. (9). This lack of effect may be attributable to multiple factors. Firstly, executive function constitutes a complex process involving planning, cognitive flexibility, and inhibition, potentially relying upon more dispersed and intricate neural networks that are less susceptible to the localised modulatory effects of standard tDCS protocols. Secondly, the TMT tests possess multiple dimensions: TMT-A primarily assesses information processing speed and visual attention, whereas TMT-B places greater emphasis on task-switching ability and working memory. The failure of tDCS to significantly enhance performance on both subtests suggests it may be insufficiently capable of modulating these core cognitive processes (37).

### Strengths and limitations

This paper innovatively deconstructs cognitive function into four distinct domains: global cognition, visuospatial processing, executive function, and attention. It reveals the “domain-specific” effects of tDCS. Adhering to the PRISMA guidelines and being pre-registered in PROSPERO, the study exhibited low heterogeneity between studies, enhancing the reliability of the findings. However, this systematic review and meta-analysis has several limitations. Firstly, the number of studies included was small for certain cognitive domains (particularly attention, with only two studies, and executive function, with only four studies), and the total sample size was limited. This reduces the statistical power of tests and compromises the reliability of conclusions in these domains. Secondly, quality assessments revealed that some studies posed an “unknown risk” with regard to aspects such as random sequence generation, allocation concealment and blinding, which could affect the authenticity of the pooled results. Thirdly, while the cognitive assessment scales used (e.g., MMSE and MoCA) are standardised tools, they may not be sensitive enough to detect the subtle improvements induced by tDCS. Furthermore, there is a lack of follow-up data regarding long-term efficacy.

### Limitations of cognitive domain measurements

A major limitation of this meta-analysis is that, although a broad range of cognitive constructs were examined, a narrow range of tests were used. For example, executive function was assessed solely using the Trail Making Test, which does not comprehensively evaluate subdomains such as inhibition, planning, or cognitive flexibility. Attention was assessed using only the Digit Span test, which primarily measures attention span and working memory rather than sustained, selective or divided attention. Visuospatial abilities were assessed using the Clock Drawing Test, which is a screening tool rather than a specialised battery of tests for visuospatial abilities. Consequently, the domain-specific findings of this study should be regarded as hypothesis-generating rather than conclusive. Furthermore, the small number of studies on attention (k = 2) and executive function (k = 4) limits statistical power and increases the risk of Type II errors for executive function and Type I errors for attention. Future randomised controlled trials (RCTs) should use validated, multi-test neuropsychological test batteries (e.g., CANTAB or D-KEFS) for each target cognitive domain, and calculate the appropriate sample size using *a priori* power analysis.

## Conclusion

This meta-analysis provides robust evidence that tDCS improves global cognition in patients with AD and MCI as measured by the MMSE and MoCA. Preliminary evidence suggests possible benefits for visuospatial ability, but findings for attention and executive function remain inconclusive due to the small number of studies, limited sample sizes, and reliance on single neuropsychological tests per domain. tDCS remains a well-tolerated, non-pharmacological intervention with potential clinical utility for global cognitive enhancement. However, before concluding that tDCS has genuine domain-selective effects, future studies must employ adequately powered designs with multi-test, domain-specific neuropsychological batteries and longer-term follow-up assessments.

## Data Availability

The original contributions of this study are detailed within the article. For further inquiries, please contact the corresponding author.
